# PET/MR versus PET/CT for locoregional staging of oropharyngeal squamous cell cancer

**DOI:** 10.1177/02841851221140668

**Published:** 2022-12-04

**Authors:** Lennart Flygare, Secil Telli Erdogan, Karin Söderkvist

**Affiliations:** 1 Department of Radiation Sciences, Diagnostic Radiology, Umeå University, Umeå, Sweden; 2 Department of Radiation Sciences, Oncology, Umeå University, Umeå, Sweden

**Keywords:** Head and neck cancer, positron emission tomography/magnetic resonance imaging, positron emission tomography/computed tomography, 18F-FDG, cancer staging

## Abstract

**Background:**

The value of fluorine-18-fluorodeoxyglucose positron emission tomography/computed tomography (FDG-PET/CT) for TN staging in head and neck cancer (HNC) has been proven in numerous studies. A few studies have investigated the value of FDG-PET/magnetic resonance imaging (MRI) in the staging of HNC; the combined results indicate potential for FDG-PET/MRI, but the scientific evidence remains weak.

**Purpose:**

To compare performance of FDG-PET/CT and FDG-PET/MRI for locoregional staging in patients with oropharyngeal carcinomas.

**Material and Methods:**

Two radiologists independently of each other retrospectively reviewed primary pre-therapeutic FDG-PET/CT and FDG-PET/MRI examinations from 40 individuals with oropharyngeal carcinomas. TN stage and primary tumor size were noted. The results were compared between observers and modalities and against TN stage set at a multidisciplinary conference.

**Results:**

For nodal staging, PET/MRI had slightly higher specificity and accuracy than PET/CT for the most experienced observer. Both methods demonstrated excellent sensitivity (≥ 0.97 and 1.00, respectively), as well as high negative predictive values (≥ 0.95 and 1.00, respectively). No significant differences were found for tumor staging or measurement of maximum tumor diameter. There was a weak agreement (κ = 0.35–0.49) between PET/CT and PET/MRI for T and N stages for both observers. Inter-observer agreement was higher for PET/MRI than for PET/CT, both for tumor staging (κ = 0.57 vs. 0.35) and nodal staging (κ = 0.69 vs. 0.55). The agreement between observers was comparable to the agreement between methods.

**Conclusion:**

PET/MRI may be a viable alternative to PET/CT for locoregional staging (TN staging) and assessment of maximal tumor diameter in oropharyngeal squamous cell cancer.

## Introduction

The value of fluorine-18-fluorodeoxyglucose positron emission tomography/computed tomography (FDG-PET/CT) for TN staging in head and neck cancer (HNC) has been proven in numerous studies (1–4). Until recently, only a few studies had investigated the value of FDG-PET/magnetic resonance imaging (FDG-PET/MRI) in the staging of HNC, with equivocal results (5–12). In recent years, several new studies have highlighted the potential of PET/MRI as an alternative to PET/CT (13–20). The scientific evidence, however, remains weak.

One drawback of FDG-PET/MR is that whole-body MRI is more technically challenging and requires more time to perform than whole body FDG-PET/CT (21). In a recent study, we found that for small HNC tumors (T1–T2) with a clinically negative neck, the risk for distant metastasis is extremely low and no squamous cell carcinomas (SCCs) in a cohort of 335 cases had set subphrenic metastases (22). On the other hand, whole-body imaging with PET/CT in early stage HNC can trigger excessive investigations of benign abdominal lesions that can cause delays in treatment (23,24). Therefore, an FDG-PET/MRI scan of the head and neck region combined with a CT scan of the thorax may be a sufficient alternative for TNM staging in such patients on the premise that the TN staging property of FDG-PET/MRI compares to or supersedes that of FDG-PET/CT. The superior soft tissue visualization of MRI offers advantages, especially in the suprahyoid region (25). The superiority of FDG-PET/MRI was also proposed in a comparative study on nasopharyngeal carcinomas, conducted with non-contrast-enhanced FDG-PET/CT versus contrast-enhanced FDG-PET/MRI (26). The aim of the present study was to compare locoregional TN staging as well as assessments of tumor diameter between pretherapeutic FDG-PET/CT and FDG-PET/MRI in a cohort of patients with oropharyngeal squamous cell carcinomas (OPSCCs).

## Material and Methods

In a retrospective setting, primary pretherapeutic FDG-PET/CT and FDG-PET/MRI examinations of 40 patients with histologically proven OPSCCs were reviewed by two radiologists (LF and SE) independently of each other. The radiologists had 25 and 5 years of experience of head and neck radiology, respectively.

### Participants

**Fig. 1.**
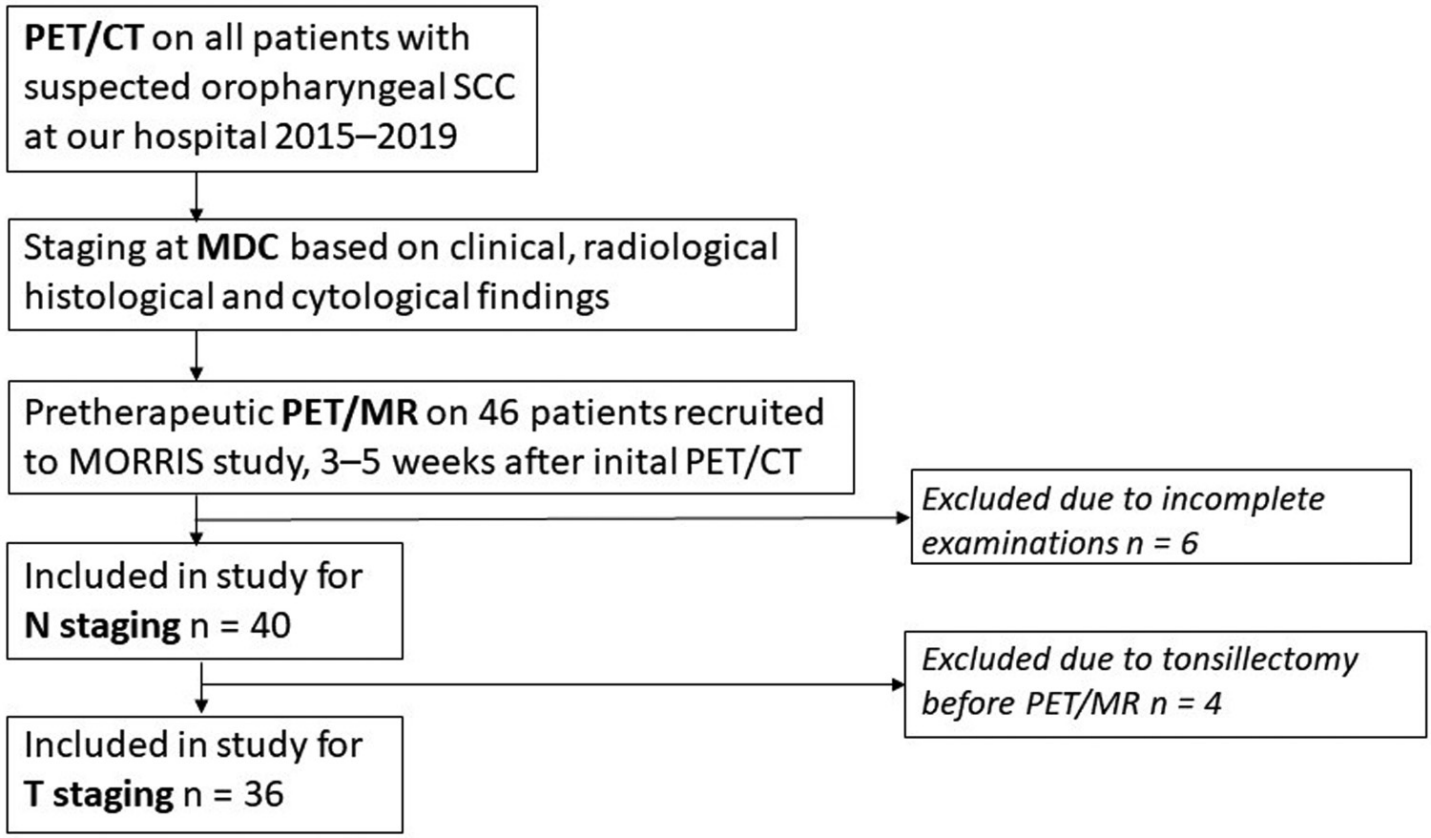
Flow chart of study design. MDC, multidisciplinary conference; SCC, squamous cell carcinoma.

The participants consisted of a cohort of patients with OPSCCs enlisted in the ongoing prospective clinical trial Multimodal Monitoring of Radiotherapy Response in Squamous Cell Cancer (MORRIS; NCT02379039). The aim of the MORRIS trial is the prediction of short- and long-term outcome after radiotherapy in SCC. The first 46 patients with OPSCC recruited in the MORRIS trial were evaluated for inclusion in the present project. Six patients were excluded due to incomplete radiology. A total of 40 patients (32 men, 8 women; mean age 64.1 years; age range = 40–84 years) were included in the analysis (Fig. 1 and Supplementary Table 1). The T classifications were in the range of T2–T4a, and the N classifications were in the range of N0–N3. In total, 27 patients were HPV-positive, and eight patients were HPV-negative. HPV status was not known in five patients; these patients were regarded as HPV-negative when setting the N classification.

### Image acquisition

PET/CT was acquired on a Discovery 690 64-slice PET/CT scanner (GE Healthcare, Chicago, IL, USA), after 6 h of fasting. Imaging was carried out 1 h after intravenous administration of 4 MBq/kg body weight of 18F-FDG. The PET acquisition was made separately for the head and neck region, with high-resolution reconstruction (SHARP) in the head and neck area. A standard acquisition protocol and reconstruction were applied for the rest of the body. After PET sampling, a diagnostic contrast-enhanced CT was performed of the thorax, abdomen, and neck. The neck was scanned separately with a smaller field of view to enhance in-plane resolution. The CT scans were performed with the patient in the same position as during the initial PET, enabling co-registered volumes. The total scanning time was 45 min.

The day after the PET/CT examination, biopsies were taken from primary tumor sites for histological analysis and assessment of HPV status by p16 expression with immunohistochemistry. The neck was examined with ultrasound, and ultrasound-guided fine-needle aspiration cytology (US-FNAC) was performed on all neck levels with nodes deemed pathological based on ultrasound or PET/CT.

Within one week from the PET/CT examination, diagnosis and TNM stage were set at a multidisciplinary conference (MDC), based on all available information from clinical and radiological findings, histopathology of primary tumor biopsies including assessment of HPV status and cytology of US-FNAC from regional lymph nodes.

PET/MRI scanning was always performed after the MDC but before the patient received any other treatment, on average 27 days (range = 17–45 days) after the PET/CT. The PET/MRI scan was carried out on a SIGNA™ PET/MRI 3.0-T scanner (GE Healthcare, Chicago, IL, USA) after 6 h of fasting. Imaging was carried out 1 h after intravenous administration of 4 MBq/kg body weight of 18F-FDG.

The PET/MRI examinations consisted of T1-weighted (T1W), T2-weighted (T2W), and diffusion-weighted imaging (DWI) as well as PET and fusion images. Details on MRI parameters are presented in Supplementary Table 2. From a stack of dynamic contrast-enhanced T1W sequence images, a stack of synthetic contrast-enhanced T1W images was calculated and saved. All the images were pseudonymized and patient information blinded for the observers.

Before the readings commenced, the observers calibrated their performance by consensus TN staging on five FDG-PET/CT scans from another patient cohort. The TN staging in this study followed the 8th edition of the UICC TNM classification of malignant tumors (27).

### Image interpretation

Observer 1 first read all the PET/MRI scans, and after a period of one month read the PET/CT scans. Observer 2 did the opposite. Primary tumor localization, T stage, and largest diameter of primary tumor were noted as well as occurrence of nodal metastases on each level of the neck, as defined by the AJCC (28). The largest diameter of the largest node in each level was noted.

As a third step, 12 cases with a difference in tumor location or considerable difference in tumor staging between PET/CT and PET/MRI were reviewed in consensus. Four patients had undergone surgical intervention with tonsillectomy or extensive biopsies of the primary tumor site between PET/CT and PET/MRI examination, and those cases were excluded from the results of the PET/MRI-based tumour staging (Fig. 1).

The N stage for each observer's readings was set according to the 8th edition of the UICC TNM classification (27).

The outcome from the readings was compared in terms of agreement between FDG-PET/MRI and FDG-PET/CT as well as agreement with the final TN stage as set at the MDC, which served as the gold standard. Finally, intra- and inter-observer variation of the different modalities was assessed.

### Statistical analysis

SPSS Statistics software version 27.0.1.0 (IBM Corp., Armonk, NY, USA) was used for the statistical analyses. Interrater reliability was calculated as Cohen's Kappa index; and the Wilcoxon matched-pair signed-rank test was used to test for systemic differences between observers or methods in staging and tumor size measurements. *P* < 0.05 was considered statistically significant.

Sensitivity, specificity, predictive values, and likelihood ratios were calculated for nodal staging with MDC consensus as the gold standard and neck side as the observational unit. Thus, there were 80 observations in 40 patients per observer and modality.

The calculation of *P* values for differences in diagnostic performance were performed using R 4.1.1 (R Core Team, Vienna, Austria) and the DTComPair version 1.0.3 (Christian Stock, Thomas Hielscher, 2014) package. For sensitivity, specificity, and accuracy, McNemar’s test of two binary diagnostics in a paired study design was used to test for systemic differences between observers or methods. Tests for differences in predictive values were carried out as proposed by Moskowitz and Pepe (29) and for the likelihood ratio as proposed by Gu and Pepe (30).

### Ethical approval

Approval from the institutional review board was obtained from the regional ethics committee before the study (EPN 2015/117-31).

## Results

The clinical features of the cohort are presented in Supplementary Table 1. For the mapping of nodal neck metastases, both PET/CT and PET/MRI demonstrated good accuracy, excellent sensitivity, and high negative predictive values (NPVs) (Tables 1 and 2). Specificity was slightly higher for PET/MRI than for PET/CT. PET/MRI also exhibited a higher accuracy than PET/CT. The differences between methods in specificity and accuracy, in favor of PET/MR, were present for both observers, but were statistically significant only for the more experienced observer 1 (Tables 1 and 2). For PET/MRI, there were significant differences between observers in specificity (*P* = 0.002) and in accuracy (*P* = 0.009).

**Table 1.** Findings of metastatic lymph nodes in 40 patients with OSCC on PET/MRI and PET/CT by two observers.

	Observer 1		Observer 2	
	MDC+	MDC–	MDC+	MDC–
PET/CT+	36	16	35	23
PET/CT–	0	28	1	21
PET/MRI+	35	7	35	17
PET/MRI–	1	37	1	27
Total	36	44	36	44

Observational unit is neck side, i.e. two observations per patient and 80 observations per observer.

CT, computed tomography; MDC, multidisciplinary conference; MRI, magnetic resonance imaging; OSCC, oropharyngeal squamous cell carcinoma; PET, positron emission tomography.

**Table 2.** Diagnostic performance for mapping of metastatic neck nodes by PET/CT and PET/MRI as compared to MDC consensus based on all available clinical, radiological, and histopathological findings including US-FNAC, in 40 patients with oropharyngeal SCC.

	Observer 1			Observer 2		
	PET/CT	*P* value	PET/MRI	PET/CT	*P* value	PET/MRI
Sensitivity	1	0.317	0.97	0.97	1.000	0.97
Specificity	0.64	0.007*	0.84	0.48	0.109	0.61
PPV	0.69	0.009*	0.83	0.60	0.117	0.67
NPV	1	0.317	0.97	0.95	0.862	0.96
PLR	2.75	0.008*^†^	6.11	1.86	0.118	2.52
NLR	0	0.744	0.03	0.06	0.860	0.05
Accuracy	0.8	0.043*	0.9	0.7	0.211	0.78

One observation per neck side. Data on which calculated statistics are based are presented in Supplementary Tables 3–6.

CT, computed tomography; MDC, multidisciplinary conference; MRI, magnetic resonance imaging; NLR, negative likelihood ratio; NPV, negative predictive value; OSCC, oropharyngeal squamous cell carcinoma; PET, positron emission tomography; PLR, positive likelihood ratio; PPV, positive predictive value; SCC, squamous cell carcinoma; US-FNAC, ultrasound-guided fine-needle aspiration cytology

*Statistically significant.

^†^ Due to the number of frequencies equal to zero for observer 1, *P* values for positive and negative likelihood ratio were based on non-parametric percentile bootstrapping of the difference between PET/CT vs. PET/MRI supported by R 4.1.1 (R Core Team, 2018), the boot (v1.3.28), and boot.pval (v0.4) packages.

Negative likelihood ratios were excellent for both methods (0.00–0.05), whereas positive likelihood ratios were moderate (1.86–6.11) (Table 2).

No statistically significant differences in tumor staging nor in measurement of maximum tumor diameter were found between modalities or observers.

There was a weak agreement (κ = 0.35–0.49) between PET/CT and PET/MRI for T and N stages for both observers. Inter-observer agreement was higher for PET/MRI than for PET/CT, both for tumor staging (κ = 0.57 vs. 0.35) and for nodal staging (κ = 0.69 vs. 0.55) (Tables 3 and 4). For both observers, there was lower agreement between PET/MRI and MDC for tumor staging (minimal, κ = 0.25–0.28) than between PET/CT and MDC (weak, κ = 0.47–0.53).

**Table 3.** Interrater reliability between PET/MR and PET/CT or Observers 1 and 2 and MDC for tumour classification according to UICC TNM Classification of Malignant Tumours. 8th ed (27) in 36 patients with oropharyngeal carcinoma.

Evaluation	Agreement (% of observations)	CoheńsKappa	95% CI
PET/MR **/** PET/CT			
Obs 1	61.1	0.45	0.23–0.67
Obs 2	61.1	0.40	0.17–0.64
PET/CT			
Obs 1/Obs 2	55	0.35	0.13–0.58
Obs 1/MDC	62.5	0.47	0.26–0.67
Obs 2/MDC	67.5	0.53	0.32–0.73
PET/MR			
Obs 1/Obs 2	70	0.57	0.37–0.77
Obs 1/MDC	50	0.28	0.06–0.50
Obs 2/MDC	50	0.25	0.02–0.48

CI = Confidence interval, Obs = Observer, MDC = Multidisciplinary conference

**Table 4.** Interrater reliability between PET/MR and PET/CT or Observers 1 and 2 for nodal classification according to UICC TNM Classification of Malignant Tumours. 8th ed (27) in 40 patients with oropharyngeal carcinoma.

Evaluation	Agreement (% of observations)	Coheńs Kappa	95% CI
PET/MR **/** PET/CT			
Obs 1	62.5	0.49	0.30–0.69
Obs 2	55	0.35	0.14–0.56
PET/CT			
Obs 1/Obs 2	67.5	0.55	0.33–0.74
Obs 1/MDC	60	0.48	0.31–0.66
Obs 2/MDC	45	0.30	0.14–0.46
PET/MR			
Obs 1/Obs 2	77.5	0.69	0.52–0.86
Obs 1/MDC	70	0.60	0.43–0.78
Obs 2/MDC	50	0.34	0.16–0.52

CI = Confidence interval, Obs = Observer, MDC = Multidisciplinary conference

## Discussion

In the present study, we found statistically significant differences between PET/CT and PET/MRI in specificity and accuracy in the nodal staging of HNC for one of two observers. The difference was in favor of PET/MRI, and these findings differ from previous studies that have failed to demonstrate any difference between the methods (5,6,9,17,19,31–33). Most previous studies, however, experienced small samples or used observer consensus as the gold standard. The scientific evidence for using PET/MRI in the staging of HNCs is scarce, and the results from this study add evidence to the notion that PET/MRI is a valid replacement for PET/CT in such cases.

It should be emphasized that although both observers in this study obtained slightly better results for nodal staging with PET/MRI than with PET/CT in all diagnostic parameters except sensitivity, only the more experienced observer 1 achieved statistically significant differences. MRI has been shown to be superior to CT for obtaining excellent soft tissue contrast and is less sensitive than CT for artifacts from dental hardware. Conventional MRI sequences are also superior to CT for a variety of findings that influence the therapeutic choice such as skull base invasion, perineural spread, detection of retropharyngeal nodes, extranodal spread in metastatic neck nodes, and vascular and lymphatic invasion (21). However, MRI may be more dependent than CT on observer experience as a plethora of technical parameters and possible artefact sources need to be considered in the image interpretation.

In general, there was a larger difference between observers than between methods (Table 1). The inter-observer variation was higher for tumor staging than for nodal staging for both modalities. This seems logical, as radiological T staging of oropharyngeal tumors is dependent on involvement of delicate anatomical structures and may be more complicated than nodal staging, which is primarily based on size, multiplicity, and bilaterality (27).

**Fig. 2.**
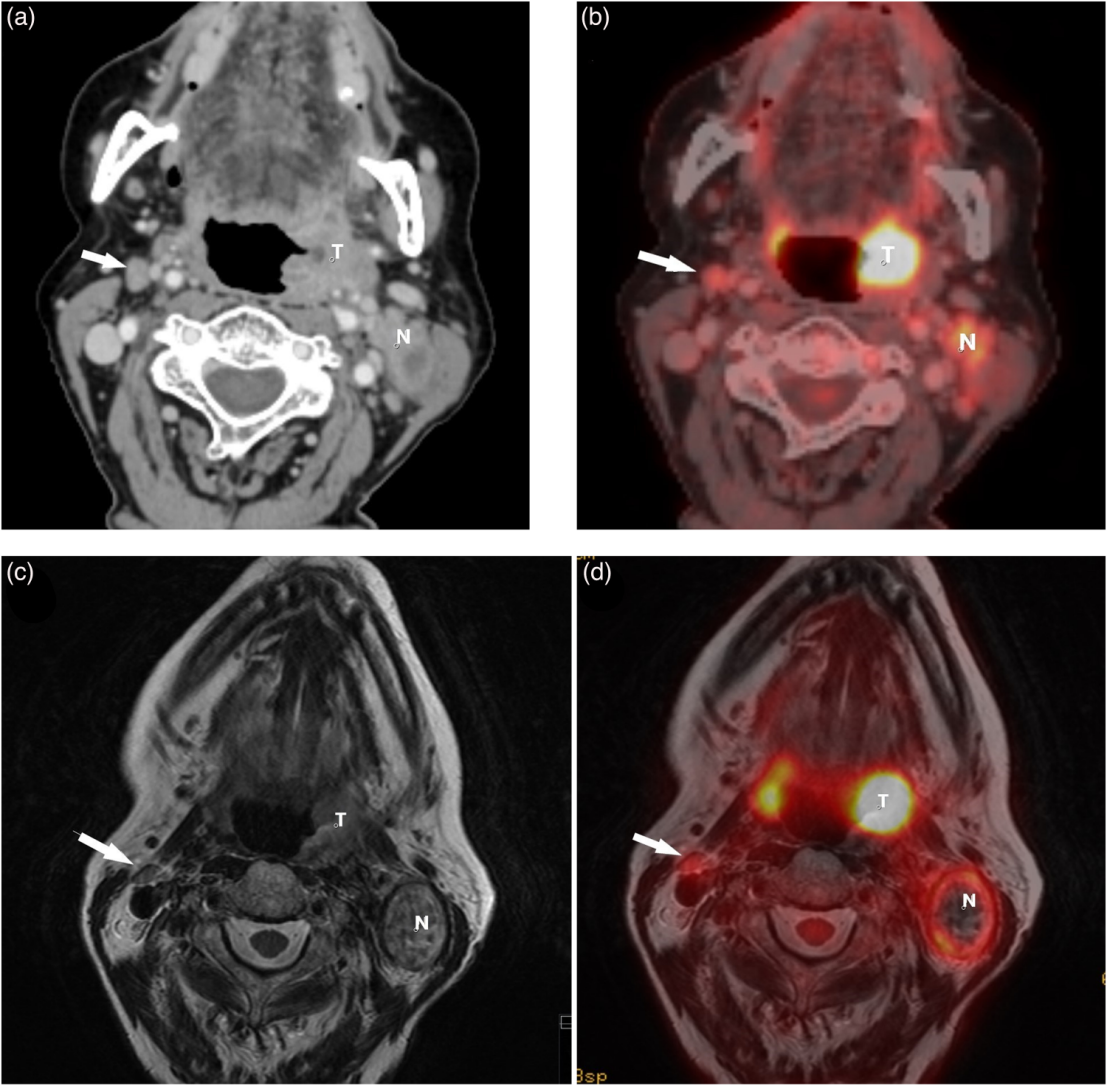
A patient with a SCC in the left tonsil and ipsilateral metastasis. The right-sided node (arrow) was judged as metastatic on PET/CT (false positive) but not on PET/MRI (true negative). US-FNAC of the node revealed no malignant cells, and the node was at considered reactive at the MDC. Arrow = contralateral reactive node. (a) CT scan reveals slightly enlarged, rounded right-sided node (arrow). (b) A FDG-PET/CT hybrid image shows the node (arrow) has an elevated FDG-tracer uptake (SUV_max_ 5.4). (c) On T2W MRI, the node (arrow) has a normal fusiform appearance. (d) FDG-PET/MRI hybrid image demonstrates mild FDG-tracer uptake (SUV_max_ 4.5) in the node (arrow). CT, computed tomography; MDC, multidisciplinary conference; MRI, magnetic resonance imaging; N, metastatic ipsilateral node; PET, positron emission tomography; SCC, squamous cell carcinoma; T, tumor; T2W, T2-weighted; US-FNAC, ultrasound-guided fine-needle aspiration cytology.

PET/MRI had a slightly lower agreement with the MDC for tumor staging than had PET/CT. This may be due to inclusion bias, as the results from PET/CT formed part of the base for the gold standard, that is, TN stage set at the MDC. Since the difference was not statistically significant, we believe PET/MRI to be at least equal to PET/CT for tumor staging of OPSCCs. Furthermore, PET/MRI performed slightly better than PET/CT for nodal staging, both in terms of interrater reliability as well as in specificity and accuracy (Tables 1 and 2, Fig. 2). As our gold standard for nodal staging also relied on the results of US-FNAC and thus probably is closer to the truth, we believe these findings speaks in favor of PET/MRI.

Samolyk-Kogaczeska and co-workers (16), using a histopathological reference standard, reported better agreement in T staging and higher specificity, sensitivity, positive predictive values (PPV) and NPVs of lymph nodes evaluation by PET/MRI than CT imaging. Our findings indicate that the same may be valid for PET/MRI when compared to PET/CT, as in our study, inter-observer agreement for N staging was substantial for PET/MRI (κ = 0.69) and moderate for PET/CT (κ = 0.55). PET/MRI also demonstrated slightly higher sensitivity, specificity, PPV, and NPV for nodal staging than PET/CT, albeit the differences were not statistically significant.

There are a number of known causes for both false-positive and false-negative evaluations described in the literature. For instance, necrotic nodes sometimes are without elevated FDG uptake and therefore overlooked by the radiologist. Furthermore, small metastatic nodes can be without elevated FDG uptake or suspicious morphological features and may be missed with any imaging modality (21). Many non-metastatic nodes, on the other hand, may show elevated FDG uptake or suspicious features due to inflammatory reaction. In our material, the false-negative findings were scarce whereas the false-positive fraction was significant (Table 1). The sensitivity was higher and the specificity was slightly lower compared to early studies for both PET/CT and PET/MRI (5,11,34) but were comparable to more recent studies (17,19). This is in line with the tradition at our institute where high sensitivity is favored over specificity since the results from PET imaging are routinely validated by US-FNAC.

**Fig. 3.**
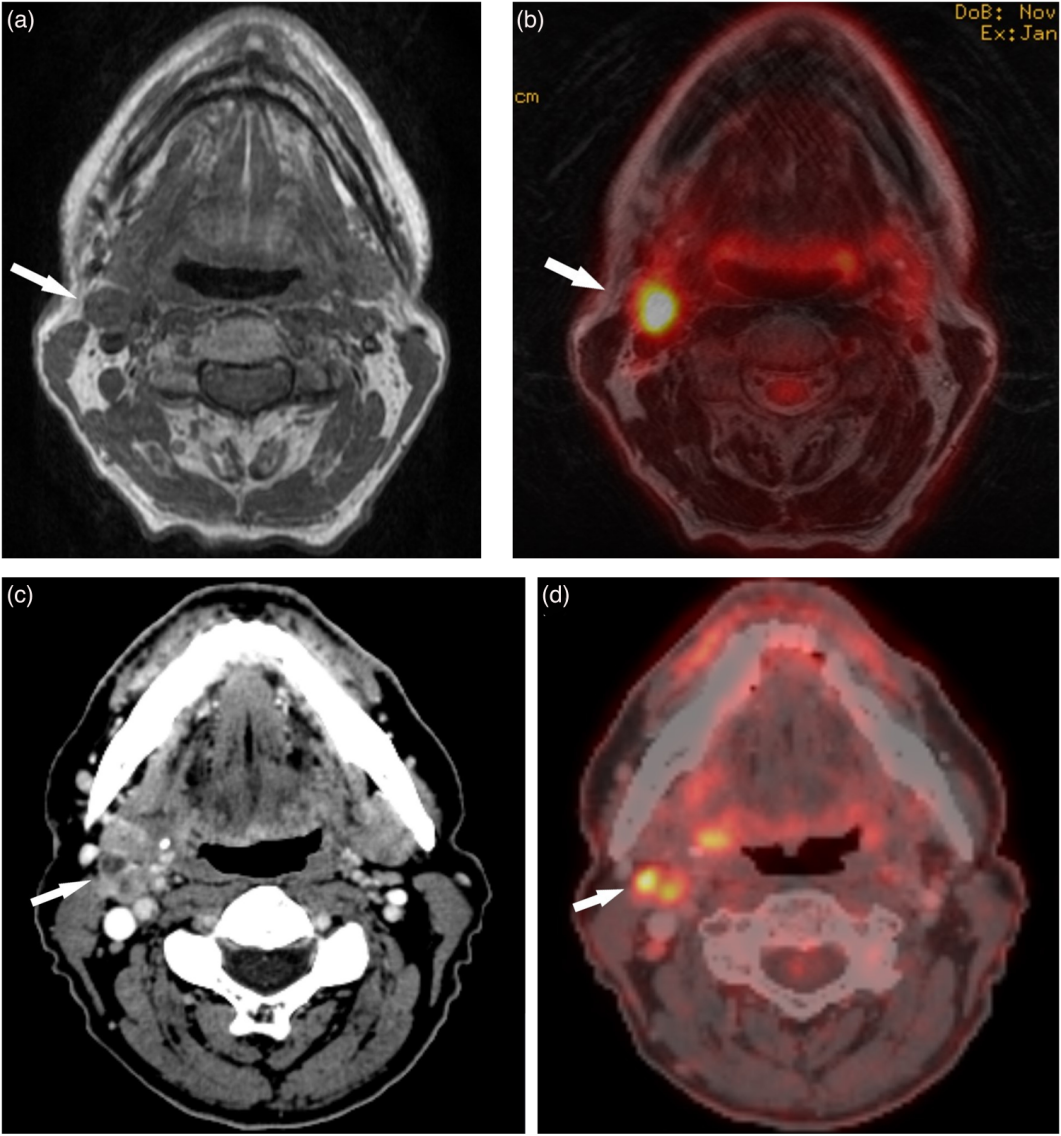
A patient with SCC in the right tonsil and unilateral metastatic nodal conglomerate. (a, b) T1W and T2W PET/MRI hybrid image demonstrate a metastatic node (arrows) with elevated FDG uptake (SUV_max_ 9.6) in the right jugulodigastric area. (c, d) CT and PET/CT hybrid image reveals a conglomerate of metastatic nodes (arrows) with elevated FDG uptake (SUV_max_ 9.6) in the same position. The PET/CT findings were later confirmed by US-FNAC. CT, computed tomography; MRI, magnetic resonance imaging; PET, positron emission tomography; SCC, squamous cell carcinoma; T1W, T1-weighted; T2W, T2-weighted; US-FNAC, ultrasound-guided fine-needle aspiration cytology.

The pattern of overstaging of nodes between PET/CT and PET/MRI was similar, although there were slightly fewer false-positive findings with PET/MRI. Understaging is, in our point of view, more worrisome since false-negative evaluations may have dire consequences for the patient whereas overstaging can be dealt with by follow-up US-FNAC. In two cases, staged as N2b at the MDC, both observers understaged nodes with PET/MRI only. One case was judged with PET/MRI to be a single node (N1), whereas both PET/CT and ultrasound revealed a conglomerate of nodes at the same location, upstaging it to N2b (Fig. 3). In the other case, PET/CT indicated multiple suspicious nodes, and stage N2b was set at the MDC. Even, so the results from US-FNAC were positive only in one location and, in our opinion, N1, as indicated by both PET/MRI and US-FNAC, would be the proper staging. We regard this as a case of incorporation bias.

Another factor speaking in favor of PET/MRI is its inherently lower radiation dose compared with that of PET/CT. One could argue that the dose from PET/CT is negligible compared to the radiation treatment these patients will undergo. However, not all patients examined with PET/CT will undergo subsequent radiation therapy, as robot-assisted surgery is introduced for early stages of oropharyngeal cancer. We argue that the radiation dose from the diagnostic examination does matter, even in patients with a suspected HNC tumor that subsequently turns out to be benign.

The present study has some limitations. First, the gold standard was TN staging as set in an MDC. This staging was partly based on the results of the initial PET/CT, leading to incorporation bias that could favor the estimated accuracy of the PET/CT, especially in T staging. A better gold standard would be histologic evidence from surgical specimens. However, as all patients with OPSCCs at our institute are primarily treated with radiation therapy, surgical validation was not available, and the TN staging set at the MDC was as good a gold standard as we could achieve. The US-FNAC of the neck nodes were performed as a clinical examination and it was not possible in retrospect to pinpoint the exact node biopsied. Therefore, we used the neck side for further analyses.

Biopsies of primary tumors and US-FNAC of neck nodes were performed between PET/CT and PET/MRI, which could possibly skew the results from the PET/MRI examination.

The limited number of patients included incurs several weaknesses. First, it reduces the validity of the statistical results. The small sample size also prevents any reliable comparison between HPV-positive and HPV-negative OPSCC staging. It can also be questioned how reflective of a normal population this small patient cohort is. There was a skewness towards more men and HPV-positive disease in the cohort (Supplementary Table 1). While the p16 distribution is reflective of the spectrum of patients at our institute, the strong male predominance is not. Due to the inclusion criteria in the underlying MORRIS study, there was also a skewness towards advanced primary tumors.

The PET/MRI examinations were performed using a restricted research protocol. A full clinical protocol would also include T2W fat-saturated images, possibly more sensitive for nodes as well as a T1W spin echo sequence with gadolinium contrast enhancement, which we believe can result in more accurate tumor delineation. On the other hand, in a previous study, Pyatigorskaya et al. (15) found no added value in gadolinium contrast for tumor delineation in head and neck MRI.

In conclusion, PET/MRI may serve as a valid replacement for PET/CT for the loco-regional staging of HNC, as it equaled PET/CT for tumor staging and in sensitivity for identifying nodal positive necks. PET/MRI was more specific than PET/CT, albeit only for the more experienced observer. Observer experience had, in general, as high an impact on diagnostic outcome as the choice between PET/CT and PET/MRI.

## Supplemental Material

sj-doc-1-acr-10.1177_02841851221140668 - Supplemental material for PET/MR versus PET/CT for locoregional staging of oropharyngeal squamous cell cancer Click here for additional data file. Supplemental material, sj-doc-1-acr-10.1177_02841851221140668 for PET/MR versus PET/CT for locoregional staging of oropharyngeal squamous cell cancer by Lennart Flygare, Secil Telli Erdogan and Karin Söderkvist in Acta Radiologica

## References

[1] Kyzas PA Evangelou E Denaxa-Kyza D , et al. 18F-fluorodeoxyglucose positron emission tomography to evaluate cervical node metastases in patients with head and neck squamous cell carcinoma: a meta-analysis. J Natl Cancer Inst 2008;100:712–720. 18477804 10.1093/jnci/djn125

[2] Sun R Tang X Yang Y , et al. 18FDG-PET/CT for the detection of regional nodal metastasis in patients with head and neck cancer: a meta-analysis. Oral Oncol 2015;51:314–320. 25619735 10.1016/j.oraloncology.2015.01.004

[3] Petersen H Holdgaard PC Madsen PH , et al. FDG PET/CT in cancer: comparison of actual use with literature-based recommendations. Eur J Nucl Med Mol Imaging 2016;43:695–706. 26519292 10.1007/s00259-015-3217-0 PMC4764641

[4] Sanli Y Zukotynski K Mittra E , et al. Update 2018: 18F-FDG PET/CT and PET/MRI in head and neck cancer. Clin Nucl Med 2018;43:e439–e452. 30394934 10.1097/RLU.0000000000002247

[5] Schaarschmidt BM Heusch P Buchbender C , et al. Locoregional tumour evaluation of squamous cell carcinoma in the head and neck area: a comparison between MRI, PET/CT and integrated PET/MRI. Eur J Nucl Med Mol Imaging 2016;43:92–102. 26243264 10.1007/s00259-015-3145-z

[6] Morsing A Hildebrandt MG Vilstrup MH , et al. Hybrid PET/MRI in major cancers: a scoping review. Eur J Nucl Med Mol Imaging 2019;46:2138–2151. 31267161 10.1007/s00259-019-04402-8

[7] Platzek I Beuthien-Baumann B Schneider M , et al. PET/MRI in head and neck cancer: initial experience. Eur J Nucl Med Mol Imaging 2013;40:6–11. 23053322 10.1007/s00259-012-2248-z PMC3510405

[8] Lee SJ Seo HJ Cheon GJ , et al. Usefulness of integrated PET/MRI in head and neck cancer: a preliminary study. Nucl Med Mol Imaging 2014;48:98–105. 24900149 10.1007/s13139-013-0252-2 PMC4028474

[9] Heusch P Sproll C Buchbender C , et al. Diagnostic accuracy of ultrasound, 18F–FDG-PET/CT, and fused 18F-FDG-PET-MR images with DWI for the detection of cervical lymph node metastases of HNSCC. Clin Oral Investig 2014;18:969–978. 10.1007/s00784-013-1050-z 23892450

[10] Huellner MW . PET/MR in head and neck cancer - an update. Semin Nucl Med 2021;51:26–38. 33246536 10.1053/j.semnuclmed.2020.07.006

[11] Platzek I Beuthien-Baumann B Schneider M , et al. FDG PET/MR for lymph node staging in head and neck cancer. Eur J Radiol 2014;83:1163–1168. 24746792 10.1016/j.ejrad.2014.03.023

[12] Crimì F Borsetto D Stramare R , et al. [(18)F]FDG PET/MRI versus contrast-enhanced MRI in detecting regional HNSCC metastases. Ann Nucl Med 2021;35:260–269. 33454923 10.1007/s12149-020-01565-5

[13] Pace L Nicolai E Cavaliere C , et al. Prognostic value of 18F-FDG PET/MRI in patients with advanced oropharyngeal and hypopharyngeal squamous cell carcinoma. Ann Nucl Med 2021;35:479–484. 33575927 10.1007/s12149-021-01590-y PMC7981313

[14] Park J Pak K Yun TJ , et al. Diagnostic accuracy and confidence of [18F] FDG PET/MRI in comparison with PET or MRI alone in head and neck cancer. Sci Rep 2020;10:9490. 32528161 10.1038/s41598-020-66506-8 PMC7289810

[15] Pyatigorskaya N De Laroche R Bera G , et al. Are gadolinium-enhanced MR sequences needed in simultaneous ^18^ F-FDG-PET/MRI for tumor delineation in head and neck cancer? AJNR Am J Neuroradiol 2020;41:1888–1896. 32972956 10.3174/ajnr.A6764 PMC7661078

[16] Samolyk-Kogaczewska N Sierko E Dziemianczyk-Pakiela D , et al. Usefulness of hybrid PET/MRI in clinical evaluation of head and neck cancer patients. Cancers (Basel) 2020;12:511. 32098356 10.3390/cancers12020511 PMC7072319

[17] Slouka D Krcal J Kostlivy T , et al. A comparison of ^18^ F-FDG-PET/MRI and ^18^ F-FDG-PET/CT in the cancer staging of locoregional lymph nodes. In Vivo 2020;34:2029–2032. 32606177 10.21873/invivo.12002 PMC7439898

[18] Yeh C-H Chan S-C Lin C-Y , et al. Comparison of 18F-FDG PET/MRI, MRI, and 18F–FDG PET/CT for the detection of synchronous cancers and distant metastases in patients with oropharyngeal and hypopharyngeal squamous cell carcinoma. Eur J Nucl Med Mol Imaging 2020;47:94–104. 31606831 10.1007/s00259-019-04510-5

[19] Sekine T de Galiza Barbosa F Kuhn FP , et al. PET+MR versus PET/CT in the initial staging of head and neck cancer, using a trimodality PET/CT+MR system. Clin Imaging 2017;42:232–239. 28129606 10.1016/j.clinimag.2017.01.003

[20] Sekine T Barbosa FG Delso G , et al. Local resectability assessment of head and neck cancer: positron emission tomography/MRI versus positron emission tomography/CT. Head Neck 2017;39:1550–1558. 28500749 10.1002/hed.24783

[21] Becker M Zaidi H . Imaging in head and neck squamous cell carcinoma: the potential role of PET/MRI. Br J Radiol 2014;87:20130677. 24649835 10.1259/bjr.20130677 PMC4067029

[22] Flygare L Al-Ubaedi A Öhman W , et al. Distant metastases and synchronous malignancies on FDG-PET/CT in patients with head and neck cancer: a retrospective study. Acta Radiol 2020;61:1196–1204. 31902218 10.1177/0284185119896344 PMC7472832

[23] Wong WL . PET-CT for staging and detection of recurrence of head and neck cancer. Semin Nucl Med 2021;51:13–25. 33246535 10.1053/j.semnuclmed.2020.09.004

[24] Orme NM Fletcher JG Siddiki HA , et al. Incidental findings in imaging research: evaluating incidence, benefit, and burden. Arch Intern Med 2010;170:1525–1532. 10.1001/archinternmed.2010.317 PMC3721142 20876402

[25] Marcus C Sheikhbahaei S Shivamurthy VKN , et al. PET imaging for head and neck cancers. Radiol Clin North Am 2021;59:773–788. 34392918 10.1016/j.rcl.2021.05.005

[26] Chan S-C Yeh C-H Yen T-C , et al. Clinical utility of simultaneous whole-body 18F-FDG PET/MRI as a single-step imaging modality in the staging of primary nasopharyngeal carcinoma. Eur J Nucl Med Mol Imaging 2018;45:1297–1308. 29502310 10.1007/s00259-018-3986-3

[27] UICC TNM Classification of malignant tumours. 8th ed. Chichester: Wiley Blackwell, 2017.

[28] AJCC Cancer Staging Form Supplement.pdf. Available at: https://cancerstaging.org/references-tools/deskreferences/Documents/AJCC%20Cancer%20Staging%20Form%20Supplement.pdf (last accessed 6 April 2020).

[29] Moskowitz CS Pepe MS . Comparing the predictive values of diagnostic tests: sample size and analysis for paired study designs. Clin Trials 2006;3:272–279. 16895044 10.1191/1740774506cn147oa

[30] Gu W Pepe MS . Estimating the diagnostic likelihood ratio of a continuous marker. Biostatistics 2011;12:87–101. 20639522 10.1093/biostatistics/kxq045 PMC3006125

[31] Kuhn FP Hullner M Mader CE , et al. Contrast-enhanced PET/MR imaging versus contrast-enhanced PET/CT in head and neck cancer: how much MR information is needed? J Nucl Med 2014;55:551–558. 24491410 10.2967/jnumed.113.125443

[32] Kubiessa K Purz S Gawlitza M , et al. Initial clinical results of simultaneous 18F-FDG PET/MRI in comparison to 18F-FDG PET/CT in patients with head and neck cancer. Eur J Nucl Med Mol Imaging 2014;41:639–648. 24292211 10.1007/s00259-013-2633-2

[33] Partovi S Kohan A Vercher-Conejero JL , et al. Qualitative and quantitative performance of ^18^ F-FDG-PET/MRI versus ^18^ F-FDG-PET/CT in patients with head and neck cancer. AJNR Am J Neuroradiol 2014;35:1970–1975. 24924545 10.3174/ajnr.A3993 PMC7966242

[34] Yoon DY Hwang HS Chang SK , et al. CT, MR, US, 18F-FDG PET/CT, and their combined use for the assessment of cervical lymph node metastases in squamous cell carcinoma of the head and neck. Eur Radiol 2009;19:634–642. 18843493 10.1007/s00330-008-1192-6

