# Supplementary material for: PET/MR versus PET/CT for locoregional staging of oropharyngeal squamous cell cancer
Source: Acta Radiol. 2022 Dec 4;64(5):1865–72. doi: 10.1177/02841851221140668 (PMC10160406; doi:10.1177/02841851221140668)
Supplement: sj-doc-1-acr-10.1177_02841851221140668 - Supplemental material for PET/MR versus PET/CT for locoregional staging of oropharyngeal squamous cell cancer [file sj-doc-1-acr-10.1177_02841851221140668.doc]

Supplementary Table 1. Distribution of age, sex, HPV status by p16 expression, T- and N-stage as set on MDC in 40 patients with oropharyngeal squamous cell carcinoma**.**

|  | **n** | **Age** | **p16** | **T-stage** | | | | **N-stage** | | | |
| --- | --- | --- | --- | --- | --- | --- | --- | --- | --- | --- | --- |
|  |  | Mean(min –max***)*** | **-/+/**missing | **T1** | **T2** | **T3** | **T4** | **N0** | **N1** | **N2** | **N3** |
|  |  |  |  |  |  |  |  |  |  |  |  |
| Men | 32 | 63.2 (33-84) | 7/23/2 | 1 | 7 | 12 | 12 | 9 | 13 | 9 | 1 |
|  |  |  |  |  |  |  |  |  |  |  |  |
|  |  |  |  |  |  |  |  |  |  |  |  |
| Women | 8 | 67.9 (52-80) | 1/4/3 | 0 | 3 | 2 | 3 | 2 | 2 | 3 | 1 |
|  |  |  |  |  |  |  |  |  |  |  |  |
|  |  |  |  |  |  |  |  |  |  |  |  |
| Total | 40 | 64.1 (40-84) | 8/27/5 | 1 | 10 | 14 | 15 | 11 | 15 | 12 | 2 |
|  |  |  |  |  |  |  |  |  |  |  |  |

HPV = human papillomavirus, MDC = multidisciplinary conference.

Supplementary Table 2. Standard MR-parameters in PET/MRI of 40 patients with oropharyngeal squamous cell carcinoma, performed on a General Electric SIGNA 3.0T PET/MR.

| Sequence type | Acronym | TR (ms) | TE (ms) | Nex | Flip angle | Slice orientation | Slice thickness (mm) | Remark | Gd-contrast yes/no |
| --- | --- | --- | --- | --- | --- | --- | --- | --- | --- |
| T1W | IDEAL* | 520 - 667 | 9 | 1 | 142 | Transverse | 3 | 4-phases | no |
| T2W | FRFSE | 8555 | 99 | 1 | 142 | Transverse | 4 |  | no |
| T2W | PROPELLER | 7442 | 97 | 1.5 | 120 | Sagittal | 4 |  | no |
| EPI-DWI | FOCUS | 6000 | 70 | 4 | 90 | Transverse | 5 | b=0, 1000 | no |
| Fast SPGR | ASSET | 4.53 | 0.908 | 1 | 15 | Transverse | 5 | dynamic 70-phases | yes |

Nex = Number of averages, * = Dixon sequence, Gd = Gadolinium.

Supplementary Table 3. Findings of metastatic lymph nodes in 40 patients with oropharyngeal squamous cell carcinoma on PET/MRI by two observers. Observational unit is neck side i.e. two observations/patient and 80 observations/observer.

|  | Observer 1 PET/MRI+ | | Observer 1 PET/MRI- | |
| --- | --- | --- | --- | --- |
|  | Observer 2 PET/MRI+ | Observer 2 PET/MRI- | Observer 2 PET/MRI+ | Observer2 PET/MRI- |
| MDC+ | 34 | 1 | 1 | 0 |
| MDC- | 7 | 0 | 10 | 27 |

MDC = multidisciplinary conference

Supplementary Table 4. Findings of metastatic lymph nodes in 40 patients with oropharyngeal squamous cell carcinoma on PET/CT by two observers. Observational unit is neck side i.e. two observations/patient and 80 observations/observer.

|  | Observer 1 PET/CT + | | Observer 1 PET/CT - | |
| --- | --- | --- | --- | --- |
|  | Observer 2 PET/CT + | Observer 2 PET/CT - | Observer 2 PET/CT + | Observer 2 PET/CT - |
| MDC+ | 35 | 1 | 0 | 0 |
| MDC- | 13 | 3 | 10 | 18 |

MDC = multidisciplinary conference

Supplementary Table 5. Findings of metastatic lymph nodes in 40 patients with oropharyngeal squamous cell carcinoma on PET/MRI and PET/CT by observer 1. Observational unit is neck side i.e. two observations/patient, in total 80 observations.

|  | PET/MRI+ | | PET/MRI- | |
| --- | --- | --- | --- | --- |
|  | PET/CT+ | PET/CT- | PET/CT+ | PET/CT- |
| MDC+ | 35 | 0 | 1 | 0 |
| MDC- | 6 | 1 | 10 | 27 |

MDC = multidisciplinary conference

Supplementary Table 6. Findings of metastatic lymph nodes in 40 patients with oropharyngeal squamous cell carcinoma on PET/MRI and PET/CT by observer 2. Observational unit is neck side i.e. two observations/patient, in total 80 observations.

|  | PET/MRI+ | | PET/MRI- | |
| --- | --- | --- | --- | --- |
|  | PET/CT+ | PET/CT- | PET/CT+ | PET/CT- |
| MDC+ | 34 | 1 | 1 | 0 |
| MDC- | 13 | 4 | 10 | 17 |

MDC = multidisciplinary conference
